# Developing shared qualitative models for complex systems

**DOI:** 10.1111/cobi.13632

**Published:** 2020-12-21

**Authors:** Katie Moon, Nicola K Browne

**Affiliations:** ^1^ School of Business University of New South Wales Canberra ACT 2601 Australia; ^2^ Centre for Ecosystem Science, School of Biological, Earth and Environmental Sciences University of New South Wales Sydney NSW 2052 Australia; ^3^ School of Molecular and Life Sciences Curtin University Perth WA 6100 Australia

**Keywords:** cognitive maps, ecological modeling, influence diagrams, knowledge, perceptions, qualitative modeling, social science research methods, workshop facilitation, conocimiento, diagramas de influencia, facilitación de talleres, mapas cognitivos, métodos de investigación de ciencias sociales, modelado cualitativo, modelado ecológico, percepciones

## Abstract

Understanding complex systems is essential to ensure their conservation and effective management. Models commonly support understanding of complex ecological systems and, by extension, their conservation. Modeling, however, is largely a social process constrained by individuals’ mental models (i.e., a small‐scale internal model of how a part of the world works based on knowledge, experience, values, beliefs, and assumptions) and system complexity. To account for both system complexity and the diversity of knowledge of complex systems, we devised a novel way to develop a shared qualitative complex system model. We disaggregated a system (carbonate coral reefs) into smaller subsystem modules that each represented a functioning unit, about which an individual is likely to have more comprehensive knowledge. This modular approach allowed us to elicit an individual mental model of a defined subsystem for which the individuals had a higher level of confidence in their knowledge of the relationships between variables. The challenge then was to bring these subsystem models together to form a complete, shared model of the entire system, which we attempted through 4 phases: develop the system framework and subsystem modules; develop the individual mental model elicitation methods; elicit the mental models; and identify and isolate differences for exploration and identify similarities to cocreate a shared qualitative model. The shared qualitative model provides opportunities to develop a quantitative model to understand and predict complex system change.

## Abstract 2

Article Impact Statement: Integration of knowledge sets across disciplines improves understanding and conservation of complex systems.

## Abstract (es)

Desarrollo de Modelos Cualitativos Compartidos para Sistemas Complejos

**Resumen:**

El entendimiento de los sistemas complejos es esencial para asegurar su conservación y manejo efectivo. Es común que los modelos respalden el entendimiento de los sistemas ecológicos complejos y, por extensión, su conservación. Sin embargo, el modelado es principalmente un proceso social restringido por los modelos mentales de los individuos (es decir, un modelo interno a pequeña escala de cómo funciona una parte del mundo con base en el conocimiento, las experiencias, valores, creencias y suposiciones) y la complejidad del sistema. Para considerar tanto la complejidad del sistema como la diversidad de conocimiento sobre los sistemas complejos, diseñamos un método novedoso para desarrollar un modelo cualitativo de sistemas complejos. Desglosamos un sistema (arrecifes de coral de carbonato) en módulos más pequeños o subsistemas y cada uno representó una unidad funcional, sobre la cual es más probable que un individuo tenga mayor conocimiento integral. Este enfoque modular nos permitió obtener un modelo mental individual de un subsistema definido para el cual los individuos tuvieron un nivel más alto de confianza en su conocimiento sobre las relaciones entre las variables. Entonces, el reto fue juntar estos modelos de subsistemas para formar un modelo compartido completo del sistema entero, lo cual intentamos a lo largo de cuatro fases: desarrollar el marco de trabajo para el sistema y los módulos de los subsistemas; desarrollar los métodos de obtención para los modelos mentales individuales; obtener los modelos mentales; e identificar y aislar las diferencias para la exploración, así como identificar similitudes para crear conjuntamente un modelo cualitativo compartido. El modelo cualitativo compartido proporciona oportunidades para desarrollar un modelo cuantitativo que permita entender y predecir el cambio en los sistemas complejos.

## Introduction

Understanding complex systems is essential in ensuring their conservation and effective management. Understanding is necessary for defining relationships, interdependencies, and networks (e.g., Brandl et al. 2019); predicting how the system will respond to change (e.g., Blois et al. 2013); mapping how ecosystems will adapt, or not, to changing climatic conditions, including species migration or loss (e.g., Keppel et al. 2012); valuing and prioritizing areas for conservation and management intervention (e.g., Kennedy et al. 2013); and where necessary, for manipulating and monitoring the system (Snijders et al. 2017).

Modeling has become a common tool for supporting our understanding of complex systems and, by extension, their conservation (e.g., Baker et al. 2018). A number of methods exist to develop complex system models. For example, Voinov et al. (2018) provide a review of more than 20 participatory modeling methods, demonstrating how they can be used for different purposes, including fact‐finding, qualitative, semiquantitative, and quantitative modeling. Similarly, Kelly et al. (2013) consider design choices—purpose, data type, scale, and uncertainty treatment—in relation to 5 modeling methods: system dynamics, Bayesian networks, coupled component models, agent‐based models, and knowledge‐based models (i.e., expert systems). Other reviews consider stakeholder engagement (e.g., Elsawah et al. 2015), including human biases and beliefs (Voinov et al. 2016); expert versus novice understandings of complex systems (Hmelo‐Silver & Pfeffer 2004); planning, development, and application (Verburg et al. 2016); and decision making (Addison et al. 2013).

Despite their value, model elicitation continues to be constrained by the challenge of knowledge integration. That is, developing a model that is “flexible enough to incorporate a wide range of knowledge and operate on multiple scales and levels of resolution” (Barton et al. 2012:418; Verburg et al. 2016). Modeling complex systems will often involve knowledge across different system types (e.g., biological, chemical, economic, and social) at different spatial and temporal scales (e.g., Kelly et al. 2013). Elsawah et al. (2020) identify knowledge integration as 1 of the 8 grand challenges to modeling complex systems, that of bridging epistemologies across disciplines. The challenge arises because modeling is largely a social process, which involves individuals making decisions about how to define, construct, and operate their knowledge in a way that enables them to achieve some defined outcome (Voinov et al. 2018).

People's ability to describe a complex system is limited by their knowledge of that system and influenced by their values, beliefs, and aspirations. These elements comprise the structure component of a person's mental model, which exists in the mind as a small‐scale model of how (a part of) the world works (Johnson‐Laird 1980). The mental model also comprises a process component, which relates to the operation of the model, explaining how a person reasons, makes decisions, behaves, and filters and interprets information (Wood et al. 2012; Moon et al. 2019). Mental models are incomplete and often inconsistent representations of reality. They are also context dependent and can change over time. Eliciting an external representation of the internal mental model, therefore, captures a person's perceptions of how the system works (Jones et al. 2011).

Understanding these perceptions is critical in modeling for at least 4 main reasons. First, it can reveal areas of knowledge uncertainty, another of the grand challenges of modeling (Elsawah et al. 2020). Second, it captures a person's causal knowledge about how a system works, which can assist with quantifying complex systems (Voinov et al. 2018). Third, it can force systematic consideration of variables and their relationships to one another in a way that can often be overlooked, offering clues about counterintuitive consequences (Diffenbach 1982). Fourth, it enables learning, through processes of double (i.e., questioning the structure of the mental model) and triple (i.e., questioning the values and beliefs that underpin the model) loop learning (Biggs et al. 2011).

Ultimately, then, developing complex system models takes place, not within the natural world, but within the social world, where knowledge and knowledge claims are developed, accepted, and rejected (Pinch & Bijker 1984). In other words, knowledge is socially constructed; we actively interact with the data of our experiences, selectively filtering and framing it to create socially learned models (Pinch & Bijker 1984; Werhane et al. 2011). It is these social processes of construction that give rise to many of the challenges associated with modeling (Elsawah et al. 2020), including concerns around groupthink (i.e., “a mode of thinking that people engage in when they are deeply involved in a cohesive in‐group, when the members' strivings for unanimity override their motivation to realistically appraise alternative[s]” [Irving 1972: 9]) and implicit assumptions about how a system works (Lade et al. 2017). Thus, eliciting, illuminating, exploring, negotiating, and sharing our processes of knowledge construction is likely to bring us closer to a more accurate model of a complex system, and by extension then, a more reliable quantification of that system (Kelly et al. 2013; Voinov et al. 2018; Elsawah et al. 2020). Conservation scientists need to focus, therefore, not only on the tangible model of a complex system, but also on intangible processes of knowledge construction that are used to create that model. Doing so will contribute to overcoming some of the challenges of modeling complex systems.

### Aims and Approach

We developed a method to account for both the multidimensional and multidisciplinary nature of the complex systems, as well as the social nature of modeling complex systems. We adopted a modular approach to account for the nature of complex systems, which are comprised of tightly coupled subsystems (Van den Bossche et al. 2011). We accounted for multiple knowledge sets by eliciting individual mental models (remotely), providing opportunities to identify similarities and differences in the ways that people perceive complex systems. These individual models were then aggregated into team mental models that were subsequently used to support the development of a shared qualitative model of both the subsystem (within group) and then, collectively, the entire complex system (between groups).

**Figure 1:**
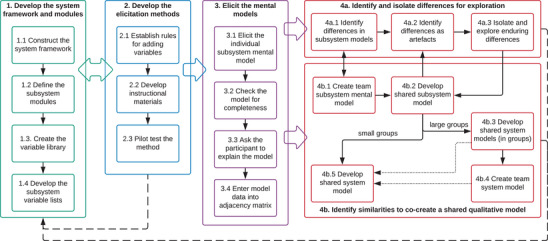
Overview of the 4 phases of the method for creating a shared qualitative model of a complex system. Appendix S1 provides additional details, including steps 1.3, 1.4, 2.1, 2.3, 3.2, and 3.4.

The development of the shared models was undertaken during a 2‐day workshop. The method involved documenting knowledge construction processes, including all supporting data and literature, and decisions and assumptions made during model development. The method was designed to enable quantitative comparison between individual mental models, as well as future conversion of the qualitative model into a quantitative model to enable monitoring, scenario planning, and prediction (Voinov et al. 2018). As such, the method requires that participants use the same researcher‐generated variables during model elicitation (see Moon et al. [2017] and Moon et al. [2019] for discussion on researcher‐ versus participant‐generated variables). The critical phases and steps of the model are outlined in Fig. 1. Additional information and details of each step are provided in Appendix S1. We used the complex system of carbonate coral reefs and associated reef islands (herein referred to as the carbonate reef system) to develop and test our method.

Carbonate coral reefs are complex systems that are characterized by diverse biological communities, dynamic abiotic environmental conditions, and multiple hierarchies of interacting biophysical components (Melbourne‐Thomas et al. 2011). They provide a number of critical ecological (e.g., habitat) and geomorphic (e.g., coastal protection) functions and generate carbonate sediments that build and maintain associated low‐lying reef islands (e.g., Kiribati Island average height of 1.8 m). These islands can support high levels of endemic biodiversity (e.g., Pilbara Islands, Western Australia) and established human populations (e.g., Maldives). To conserve and manage biodiversity and support human communities within these systems, it is necessary to understand the dynamic physical, chemical, and biological relationships that maintain reefs and their islands. Some features of these systems are particularly important to understand, such as their geoecological function and stability, which are currently threatened by climate change, ocean acidification, and rising sea level (e.g., Tuck et al. 2019).

## Develop System Framework and Subsystem Modules (Phase 1)

Complex systems can be thought of as comprising interrelated and interdependent subsystems (Ostrom 2009). Focusing on subsystems provides a number of advantages, such as permitting the in‐depth study of single subsystems; supporting the development of a common set of explanatory variables that could apply to similar subsystems in other contexts; enabling organization and ordering of the influence of different variables; assisting in identifying knowledge gaps; and providing opportunities to identify connections between subsystems to develop a complex system model (e.g., Ostrom 2009). Perhaps one of the most important advantages of focusing on subsystems is that participants are more likely to be confident in their knowledge of a defined subsystem (e.g., coral calcification and growth) compared with the entire complex system (e.g., carbonate reef system). Our method adopts a modular approach by constructing a system framework comprised of individual subsystems or modules.

### Construct System Framework of the Complex System (Step 1.1)

**Figure 2:**
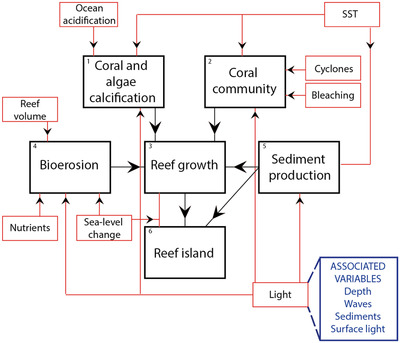
The carbonate reef system framework, which provides an overview of the dominant processes and system elements (black boxes). Red boxes represent variables (drivers) that influence 1 or more subsystem modules. The blue box is an example of additional variables that directly or indirectly influence the subsystem module(s). Variables identified are placed in appropriate variable lists (subsystem specific) and the variable library.

The system framework provides a conceptual, high‐level overview of a defined complex system through a representation of dominant processes and system elements (Fig. 2 & Table 1). Dominant processes represent a series of actions that result in a change or output. System elements are dominant components or features. In the carbonate reef system, these elements include naturally occurring entities (e.g., landscape features and organisms). System elements in social and political systems could include, for example, capital and political actors. These processes and elements will go on to become independent subsystem modules (see below) and so are intended to be high‐level categories of complexity within the system (Fig. 2).

**Table 1:** Terms and definitions applied in the development of a model of a complex system

Term	Definition
System framework	provides a conceptual overview of the complex system and includes dominant processes and elements. Drivers of change (e.g., abiotic variables) that influence these processes and elements can be identified, potentially forming the link between the system framework and subsystem modules; consists of 2 or more subsystem modules
Subsystem module	focuses on a dominant process or element that is integral to the complex system framework and should be a functioning unit of the system framework (i.e., a model in its own right with a defined output)
Mental model	individual's internal model of a system or subsystem
Team mental model	2 or more individuals’ mental models of a system or subsystem that have been elicited and aggregated
Shared qualitative system model	2 or more individuals’ agreed model of a system or subsystem; development of this model can be supported by the team mental model
Variable library	all known variables for all subsystem modules that may be included in the modeling process along with their definitions and associated units
Variable list	subsystem‐specific list of variables the modeler uses in the elicitation of their model
Processes	series of actions required to achieve an end goal, for example, biological processes include many chemical reactions that result in change and relate to a living organism
Functioning unit	defined subsystem that can be modeled separately to produce a defined and tangible output
System elements	entities, such as organisms, minerals, and chemicals
Driver variables	act as root nodes (i.e., the first node in a rooted [directed] graph from which all paths originate)
Dependent variables	high in‐degree centrality (centrality relates to the number of relationships a variable has with others)
Linkage variables	moderate to high betweenness centrality

As a first step in developing the system framework, we recommend identifying the dominant processes and system elements (Appendix S1). For example, in the carbonate reef system, it is necessary to include the reef island and the coral community as system elements. Dominant processes that affect the carbonate reef system include bioerosion, reef accretion, and sediment production (Fig. 2). The second step is to consider potential, but broad, relationships between the elements and processes (Fig. 2). For example, sediment production influences the reef island directly, but also indirectly via reef accretion. The third step is to consider any drivers of change (e.g., abiotic variables) that influence the elements or processes (e.g., Fig. 2). For example, cyclones and bleaching influence the coral community. These drivers of change may also be influenced by associated variables (e.g., Fig. 2). What is perhaps most important here is that a systematic approach be applied in the development of the system framework. Definitions and a protocol for classifying variables should be explained and described.

### Define Subsystem Module Themes (Step 1.2)

A subsystem module focuses on a dominant process or element within a subsystem that is integral to the larger system framework. The number of modules that make up the system framework depends on overall complexity of the system (i.e., potential number of variables and relationships) and available data and knowledge of the system being modeled. Developing the subsystem modules is a 2‐part process.

First, the module should represent a functioning unit (i.e., resource unit Ostrom 2009) that can produce a defined and tangible output. We define an *output* as a measurable variable that is the final product of all influences in the subsystem module and can be linked into the qualitative complex system model. Second, each subsystem module should be centered on a critical process or element. These processes and elements are often the focus of a specific discipline. For example, one of the subsystem modules focuses on the coral community (Fig. 2 & Appendix S1). Corals provide the majority of the carbonate material to reef (Perry et al. 2012; Browne et al. 2013) and island growth (Morgan & Kench 2016). How coral communities change over time and space is driven by numerous interacting physical and ecological processes and, as such, developing a subsystem module focused on this system element (i.e., an organism) is essential. The outputs for this subsystem included rates of carbonate loss, production, or accumulation with comparable measurable units (e.g., kilograms of carbonate [Appendix S1]).

## Develop the Elicitation Methods (Phase 2)

We developed materials to enable self‐elicitation of the mental model, given the resource constraints of face‐to‐face elicitation. We tested the suitability of the materials for remote elicitation to ensure that all model data could be successfully imported into an adjacency matrix (see Appendix S1). This critical phase of method development ensured that the data were suitable for modeling purposes. As such, some brief details are provided below. The decision to elicit mental models remotely should be made on the basis of project resources, the complexity of the system, and the participants’ familiarity with model elicitation.

### Develop Instructional Material (Step 2.2)

The modeling instructions included written instructions and a 10‐min instructional video (Appendix S2). The 2‐page written instructions provided an overview of the system framework as well as all 6 subsystem modules. The instructions reinforced that the modeling process was intended to elicit the participant's own mental model of how the subsystem functioned. Defined subsystem module outputs and dominant influences were provided to participants to ensure that the subsystem modules could be combined into the qualitative system model in phase 4 (Appendix S1). Instructions on the modeling process were included together with a checklist of tasks to complete. These instructions closely followed the modeling demonstration in the video. The instructional video provided an example of how to self‐elicit a mental model, developed by a social scientist who guided the modeling process and a carbonate sedimentologist who acted as the ‘expert’ in the video.

## Elicit the Individual Mental Model (Phase 3)

The intention of eliciting an individual mental model of the system was 3‐fold. First, we sought to identify those relationships that were perceived to be common among experts, providing a starting point (i.e., common ground) for the construction of the shared qualitative system model (Beers et al. 2006). Second, we elicited individual knowledge on the relationships between variables to determine which relationships could be quantitatively modeled with a sufficient degree of certainty. Participants provided data or literature to support the existence of a relationship, creating a model‐specific knowledge database. By recording this information, we were also able to identify existing knowledge gaps and determine future research needs. Third, we wanted to create the opportunity to measure differences between the individual and shared models to reveal knowledge sharing, learning, and potential effects of groupthink (Beers et al. 2006; Janis 2008). The research was conducted in line with Curtin University Ethics and approved under permit number HRE2019‐0205.

### Elicit the Individual Subsystem Mental Model (Step 3.1)

We developed the method with the intention of quantifying the final shared qualitative system model. With this goal in mind, we elicited individual subsystem mental models in a way that would provide insight into the likelihood or capacity of quantifying each relationship. As such, we asked each participant to qualify the relationships they recorded. First, participants were asked to include a directional arrow from one variable to at least one other, indicating an influence of one variable on another. This method produces a qualitative influence diagram or digraph (Cannon‐Bowers et al. 1993). Second, they were asked to classify each relationship on the basis of (1) their perception of the strength of the influence (1, weak, to 5, strong) and their confidence in their knowledge of the existence of that relationship (a, low, to c, high). This step revealed critical knowledge gaps: those relationships with low confidence ratings. See Appendix S1 for details.

Given our intention of quantifying the model, we sought an additional data set during the individual (and group, phase 4) elicitation processes. Participants were asked to document any relevant peer‐reviewed literature that supported the relationships described in the model or models. Identifying literature provides empirical data that explain or could be used to quantify relationships. These data do not necessarily enable reliable quantification of each relationship, but rather provide a starting point for quantification.

We anticipated that participants would take 1–3 h to complete the model elicitation, but time spent on the task was at their discretion.

### Ask Participants to Explain their Models (Step 3.3)

Participants were contacted to discuss their models face to face, over the phone, or via video link. The interview consisted of 15 questions across 4 themes: overview of the modeling process; model elicitation and data checking; feedback; and modeling value and benefit (Appendix S1). The overview of the modeling process provided information on how long it took participants to complete their models, their familiarity with the process, and whether they had used resources to complete the task. Questions on model elicitation and data checking provided participants with the opportunity to talk through their model, ensuring the data had been appropriately interpreted by the data analyst and highlighting whether there had been any problems with model elicitation.

Our interviews revealed that most participants took 1–3 h (30%) or 3–5 h (30%) to complete the task, although 4 people took <1 h and 1 person took a day. Participants were informed that they could use as many resources as they required for the exercise. Participants tended to use their own experiences (95%) or published work (73% others published and 59% own published) to complete their models. They were least likely to rely on unpublished work (9%) or reports (14%).

## Exploring Similarities and Differences (Phase 4)

One of the main benefits of eliciting individual mental models, using the same variables, is that it can reveal similarities and differences in how people construct their knowledge (Moon & Adams 2016; Moon et al. 2017). Similarities are important in developing consensus models, such as fuzzy cognitive maps or system dynamics models, which can be used to support decision making, enable prediction, and assess management effectiveness (e.g., Pagano et al. 2019; Pluchinotta et al. 2019). Differences in mental models are also critical to identify because they can lead to uncertainty, power struggles, disputes, poorer environmental outcomes, public opposition, lack of collaboration and coordination, and planning and implementation failures (Moon & Adams 2016). Phase 4a provides a method to identify and isolate differences in the elicited individual subsystem mental models, whereas phase 4b uses the similarities between individual models to build a team subsystem mental model in support of a shared qualitative system model. Both phases can be used simultaneously to resolve or confirm differences, as well as seek consensus (see Appendix S1 for workshop details).

### Identify and Isolate Differences for Exploration (Phase 4a)

The first step in phase 4a (step 4a.1) is to identify differences between individuals. Once data have been input into the adjacency matrix (Appendix S1), it is possible to identify differences between 2 or more participants. This process can be done manually by comparing the cells of adjacency matrices, for example, examining presence and absence of a relationship, or by examining differences between ratings for relationships. Depending on the complexity of the system, cluster analyses can also be performed to examine the extent of difference between individuals (e.g., Mathieu et al. 2005; Moon et al. 2017).

The second step in phase 4a (step 4a.2) is to identify differences as artifacts. An *artifact* is defined here as an outcome of the modeling process that results in a difference being observed where one does not actually exist. For example, 2 participants in the carbonate reef system module included the influence of water currents on coral accretion. One participant placed an arrow directly from currents to corals, whereas another placed an arrow from currents to mechanical erosion, and then to coral. The adjacency matrix records these relationships as a difference in how the system operates, where it might actually reflect the same process, but at a different level of detail.

Artifacts can be identified through the post elicitation interview (step 3.3). By asking the participants to explain their models, it becomes possible to ascertain whether variables have been used in ways that are similar, yet appear different. For instance, 2 participants included a relationship between suitable substrate and coralline algae required for coral recruitment, but in the first model, the arrow was from substrate to coralline algae and in the second model, from coralline algae to substrate. During the interviews, these participants described the same relationship (i.e., that both suitable substrate and coralline algae are required for coral recruitment), but the order in which these 2 variables interact is comparable to the chicken‐and‐the‐egg conundrum. This example illustrates differences in individual perceptions as to what should be placed first in the model and what might appear as an artifact of the social modeling process, requiring team discussion.

An important question to ask here, however, is does the artifact have any meaning (Pinch & Bijker 1984)? Given that knowledge is socially constructed, depending on the model elicitation method, artifacts could have different meaning within different (social) groups. For instance, causal processes could be perceived differently between people trained in different disciplines (e.g., ecology and geology that operate across different spatial and temporal scales). Artifacts could also increase with the number of social (or epistemic) groups involved in modeling processes. Identifying differences as artifacts is important in developing a shared qualitative system model, but it is also important to explore why they emerged.

The third step in phase 4a (step 4a.3) is to isolate enduring differences, which can be done once artifacts have been confirmed. These differences can be explored in a number of ways. First, any data or literature that corresponds with a relationship can be used to define the nature of the difference. For example, some data might be more complete or subsystem specific, enabling confirmation, rejection, or a description of uncertainty for each relationship in the model. This approach can be achieved with data collected during the individual mental model elicitation (step 3.1). Second, participants can be brought together to discuss, share, and learn from each other's models to explore and potentially resolve differences (see phase 4b).

### Identify Similarities and Explore Differences to Create a Shared Qualitative Model (Phase 4b)

**Figure 3:**
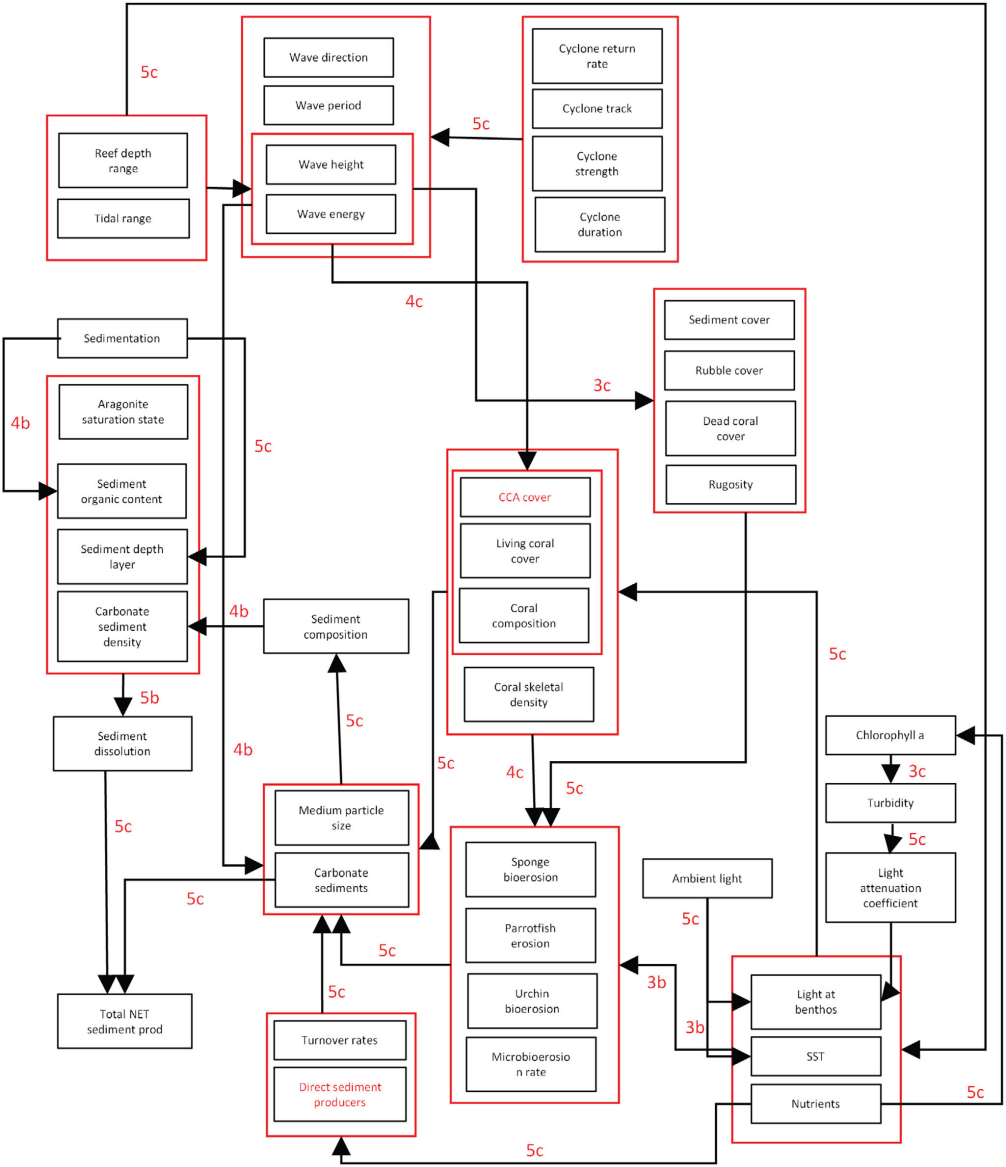
Example of an individual subsystem mental model of a carbonate coral reef system. Variables in black were provided in the variable list and library; red variables were new variables added by the participant. Each relationship was assigned a number on the basis of the participant's perception of the strength of the influence (1, weak, to 5, strong) and confidence in personal knowledge of the existence of that relationship (a, low, to c, high). (CCA, crustose coralline algae; SST, sea surface temperature).

The first step in phase 4b (step 4b.1) is to create a team mental model of the module (before the workshop). A team mental model represents 2 or more individuals’ elicited mental models (in this case, of a subsystem module) that are aggregated with one another to develop 1 summary model (Langan‐Fox et al. 2001). Prior to the workshop, 3 team subsystem mental models were created per subsystem. The first team subsystem mental model was the simplest, including only those influences identified by 3 or more participants. The second team subsystem mental model included influences identified by 2 or more people (representing a relationship in >50% of models), with colors to denote the number of people who included that relationship (e.g., green, 2 people; red, ≥3 people). The third team subsystem mental model was the most complex because it included all influences that had both a high strength and high confidence across all the individual models (Fig. 3), as well as the previous relationships highlighted. The 3 models provided participants with the opportunity to start their shared subsystem model development by focusing on the most agreed relationships across all individual models or common ground (e.g., Özesmi & Özesmi 2004; Lynam & Brown 2012).

The second step in phase 4b (step 4b.2) is to elicit a shared model of the subsystem module. A shared model represents 2 or more individuals’ perceptions of a subsystem (i.e., a module) that has been produced following shared dialogue (Moon et al. 2019). In this method, participants were brought together (day 1 of the workshop), and dialogue was supported with the team subsystem mental model (step 4b.1). Each subsystem module group was provided with a set of materials and instructions to assist in reviewing the team subsystem mental model and then developing a shared subsystem model (see Appendix S1 for example questions). It was also useful to provide copies of the individual models of all participants so they could refer to their original models during this process.

The development of the shared subsystem model can involve the group working through several iterations of a model before consensus is reached. To support this process, we provided each group with a magnetic whiteboard, several colored whiteboard markers, 50 white magnetic strips (2 × 10 cm), and a permanent marker. The magnetic strips were provided to create variables (participants would write the variable name on the strip using a permanent marker) that could easily be moved around the whiteboard. Participants were asked to use 3 colors to denote influences (i.e., arrows) between variables. Black was used for influences that all group members agreed; green was used for influences where 1 or more of the participants felt strongly that the influence was necessary and the other group participants did not feel qualified to either agree or disagree; and red was used for influences where at least 1 person disagreed. This method was included to represent the extent of sharedness in the model as well as to ensure that the modeling process could continue without a disproportionate amount of time spent on specific influences. The different influences were discussed at the end of the modeling process. Once all chosen variables and influences were finalized, group members would agree on a strength and confidence rating for each influence. Participants were given 1 full day to develop a shared subsystem model and were asked to document all decisions (Appendix S1).

The third step in phase 4b (step 4b.3) is to elicit shared qualitative models in groups. This step (day 2 of the workshop) required combining the 6 shared subsystem models into 1 qualitative system model (i.e., the complex system). For this exercise, new workshop groups were formed with a minimum of 1 person from each subsystem module group. This approach ensured that at least 1 person per group had detailed knowledge of how the shared subsystem model was developed (see Appendix S1 for a photo of a model in development).

In our study, 3 groups (6–8 people per group) developed a shared qualitative system model. Each group had a facilitator who provided some guidance for developing the model. Variable categories included driver, linkage, and dependent variables (Table 1). Therefore, the first task within the multidisciplinary groups was to identify drivers in all shared subsystem models, assess which of these drivers were common across multiple subsystem modules, and to use them to find relationships. Driver variables were the primary focus because they were the most common variables among all subsystem modules and tended to represent higher level variables that acted as root nodes. Each group developed a different method (Appendix S1). Colored magnetic strips were provided in the event they assisted with differentiating between subsystem models (Appendix S1).

**Figure 4:**
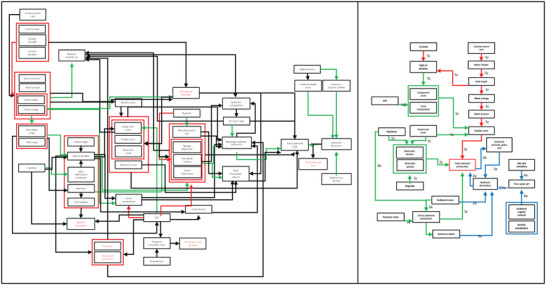
Comparison of (a) an example team subsystem model (aggregated individual mental models) and (b) shared model of a subsystem model in the carbonate reef system framework (SW carb, sea water carbonate chemistry). During a workshop designed to develop the shared model from the team model, several variables were discussed and aggregated (19 variables were reduced to 6) and relationships between variables were simplified through discussion and consensus.

The output of this step, for small groups where all participants are included in 1 group, is the final shared qualitative system model (step 4b.5) (Fig. 4). In groups of 2 or more, it is likely that different shared models will be produced, requiring an additional step or 2.

The fourth step in phase 4b (step 4b.4) is to create a team complex system model. When a large number of workshop participants have been split into smaller groups, each group develops its own shared qualitative system model. These models can be combined using data adjacency matrices (step 3.4), which are then used to create a team qualitative system model. This process is only possible if all groups have used the same variables (with some scope for new variables). When working with multiple groups, the group facilitator's role is, therefore, essential to ensure that data from each shared qualitative system model can be input into a data adjacency matrix. In our carbonate reef system model, 3 shared qualitative system models were developed, but only 2 (models 1 and 3) (Appendix S1) were used to create the team qualitative system model. Group 2 had a facilitator with limited experience in the analysis of model data and was not able to guide the development of a model that could be input into an adjacency matrix. As such, we recommend that all workshop facilitators have sufficient knowledge of how data arising from the elicitation process will be analyzed.

The fifth step in phase 4b (step 4b.5) is to develop the shared qualitative model. The shared qualitative system model is the final product of the workshop and represents the entire system being modeled. Depending on how many participants attend the workshop, cocreating the qualitative system model can either be completed in one small group (step 4b.5); directly from the (collection of) shared qualitative system models (step 4b.3), or from a team qualitative system model (step 4b.4) (Fig. 1).

When the final shared qualitative system model has to be derived from multiple shared models (options 2 and 3), 2 main pathways are possible. First, it might be possible to hold a longer workshop that allows participants to bring together their shared qualitative system models to cocreate a final model (following step 4b.2). Second, the integration of the model could be undertaken remotely, either via email or an online workshop. Given our resource constraints, we took the second approach. To assist in coordinating this process, subsystem module leaders were appointed during the workshop. We developed a team qualitative system model (distributed via email), which was reviewed by the module leaders, ensuring that it adequately represented each group's knowledge of the system. Opportunities were provided for inclusion of any feedback at this point, to be agreed by the entire group. The completed shared qualitative system model can then be finalized and distributed to all participants for member‐checking.

Given our intention to convert the shared qualitative system model into a quantitative model, future work would require identifying those relationships in the model that could be supported by empirical data and those that could not. Confidence that a relationship exists does not suggest that quantification of that relationship would necessarily be straightforward. For example, in the bioerosion subsystem, considerable evidence exists that parrotfish erode the reef structure and produce sediments, but little data exist on the amount of sediment produced per fish over time. Future applications of the method could include asking participants to provide an additional rating on data availability for each relationship.

## Contributions of the Method to Modeling Complex Systems

We developed this method in response to a need to model a complex system that demanded the integration of multiple knowledge sets across disciplines. By working collaboratively across the natural and social sciences, we were able to determine the precise needs of the modeling exercise and develop a method that works toward overcoming many of the ongoing challenges in modeling complex systems (Table 2). Our method accounts for the social construction of knowledge by documenting all decisions, assumptions, and existing data, as well as offering a modular approach that assists in dealing with the complexity of a system.

**Table 2:** Dominant challenges in modeling complex systems (Elsawah et al. 2020) and the corresponding phase of our method that seeks to assist in overcoming them

Challenge	Method
Integrating multiplicity and multidimensionality of knowledge of complex systems	elicitation of individual mental models (phase 3)
Supporting systematic consideration of causal knowledge of variables and relationships within a complex system	identification of variables related via influence diagrams (phase 1 and phase 3)
Accounting for uncertainty of relationships between variables within complex systems	specification of knowledge confidence for all relationships within the model (phase 3)
Identifying individual perceptions of a complex systems but permitting generation of a shared model	elicitation of individual mental models to support cocreation of shared qualitative system model (phase 3 and phase 4)
Improving understanding of complex systems through an exploration and integration of subsystems	creation of a modular approach to model elicitation (phase 1)
Enabling quantification of conceptual qualitative models	documentation of available evidence for all (potential) relationships (phase 3)
Developing and confirming novel model elicitation methods	development and confirmation of model elicitation method (phase 1 and phase 2)

## Supporting information

Supporting Information

Supporting Information

In the Supporting Information section at the end of the online article. The authors are solely responsible for the content and functionality of these materials. Queries should be directed to the corresponding author.Additional information is available online.

## References

[cobi13632-bib-0001] Addison PFE , et al. 2013. Practical solutions for making models indispensable in conservation decision‐making. Diversity and Distributions 19:490–502.

[cobi13632-bib-0002] Baker CM , Holden MH , Plein M , McCarthy MA , Possingham HP . 2018. Informing network management using fuzzy cognitive maps. Biological Conservation 224:122–128.

[cobi13632-bib-0003] Barton DN , et al. 2012. Bayesian networks in environmental and resource management. Integrated Environmental Assessment and Management 8:418–429. 22707420 10.1002/ieam.1327

[cobi13632-bib-0004] Beers PJ , Boshuizen HPA , Kirschner PA , Gijselaers WH . 2006. Common ground, complex problems and decision making. Group Decision and Negotiation 15:529–556.

[cobi13632-bib-0005] Biggs D , Abel N , Knight AT , Leitch A , Langston A , Ban NC . 2011. The implementation crisis in conservation planning: could “mental models” help? Conservation Letters 4:169–183.

[cobi13632-bib-0006] Blois JL , Williams JW , Fitzpatrick MC , Jackson ST , Ferrier S . 2013. Space can substitute for time in predicting climate‐change effects on biodiversity. Proceedings of the National Academy of Sciences of the United States of America 110:9374–9379. 23690569 10.1073/pnas.1220228110 PMC3677423

[cobi13632-bib-0007] Brandl SJ , et al. 2019. Coral reef ecosystem functioning: eight core processes and the role of biodiversity. Frontiers in Ecology and the Environment 17:445–454.

[cobi13632-bib-0008] Browne NK , Smithers SG , Perry CT . 2013. Carbonate and terrigenous sediment budgets for two inshore turbid reefs on the central Great Barrier Reef. Marine Geology 346:101–123.

[cobi13632-bib-0009] Cannon‐Bowers JA , Salas E , Converse S . 1993. Shared mental models in expert team decision making. Pages 221–246 in Castellan JJ , editor. Current issues in individual and group decision making. Erlbaum, Mahwah, New Jersey.

[cobi13632-bib-0010] Diffenbach J . 1982. Influence diagrams for complex strategic issues. Strategic Management Journal 3:133–146.

[cobi13632-bib-0011] Elsawah S , et al. 2020. Eight grand challenges in socio‐environmental systems modeling. Socio‐Environmental Systems Modelling 2. 10.18174/sesmo.2020a16226.

[cobi13632-bib-0012] Elsawah S , Guillaume JHA , Filatova T , Rook J , Jakeman AJ . 2015. A methodology for eliciting, representing, and analysing stakeholder knowledge for decision making on complex socio‐ecological systems: from cognitive maps to agent‐based models. Journal of Environmental Management 151:500–516. 25622296 10.1016/j.jenvman.2014.11.028

[cobi13632-bib-0013] Hmelo‐Silver CE , Pfeffer MG . 2004. Comparing expert and novice understanding of a complex system from the perspective of structures, behaviors, and functions. Cognitive Science 28:127–138.

[cobi13632-bib-0014] Irving J . 1972. Victims of groupthink. Houghton Mifflin, Boston, Massachusetts.

[cobi13632-bib-0015] Janis IL . 2008. Groupthink. IEEE Engineering Management Review 36:36–36.

[cobi13632-bib-0016] Johnson‐Laird PN . 1980. Mental models in cognitive science. Cognitive Science 4:71–115.

[cobi13632-bib-0017] Jones NA , Ross H , Lynam T , Perez P , Leitch A . 2011. Mental models: an interdisciplinary synthesis of theory and methods. Ecology and Society 16:46.

[cobi13632-bib-0018] Kelly RA , et al. 2013. Selecting among five common modelling approaches for integrated environmental assessment and management. Environmental Modelling & Software 47:159–181.

[cobi13632-bib-0019] Kennedy Emma V , et al. 2013. Avoiding coral reef functional collapse requires local and global action. Current Biology 23:912–918. 23664976 10.1016/j.cub.2013.04.020

[cobi13632-bib-0020] Keppel G , et al. 2012. Refugia: identifying and understanding safe havens for biodiversity under climate change. Global Ecology and Biogeography 21:393–404.

[cobi13632-bib-0021] Lade SJ , Haider LJ , Engström G , Schlüter M . 2017. Resilience offers escape from trapped thinking on poverty alleviation. Science Advances 3:e1603043. 28508077 10.1126/sciadv.1603043 PMC5415336

[cobi13632-bib-0022] Langan‐Fox J , Wirth A , Code S , Langfield‐Smith K , Wirth A . 2001. Analyzing shared and team mental models. International Journal of Industrial Ergonomics 28:99–112.

[cobi13632-bib-0023] Lynam T , Brown K . 2012. Mental models in human–environment interactions: theory, policy implications, and methodological explorations. Ecology and Society 17. 10.5751/ES-04257-170324.

[cobi13632-bib-0024] Mathieu JE , Heffner TS , Goodwin GF , Cannon‐Bowers JA , Salas E . 2005. Scaling the quality of teammates' mental models: equifinality and normative comparisons. Journal of Organizational Behavior 26:37–56.

[cobi13632-bib-0025] Melbourne‐Thomas J , et al. 2011. Regional‐scale scenario modeling for coral reefs: a decision support tool to inform management of a complex system. Ecological Applications 21:1380–1398. 21774437 10.1890/09-1564.1

[cobi13632-bib-0026] Moon K , Adams VM . 2016. Using quantitative influence diagrams to map natural resource managers’ mental models of invasive species management. Land Use Policy 50:341–351.

[cobi13632-bib-0027] Moon K , Blackman DA , Adams VM , Kool J . 2017. Perception matrices: an adaptation of repertory grid technique. Land Use Policy 64:451–460.

[cobi13632-bib-0028] Moon K , et al. 2019. Mental models for conservation research and practice. Conservation Letters 12:e12642.

[cobi13632-bib-0029] Morgan KM , Kench PS . 2016. Reef to island sediment connections on a Maldivian carbonate platform: using benthic ecology and biosedimentary depositional facies to examine island‐building potential. Earth Surface Processes and Landforms 41:1815–1825.

[cobi13632-bib-0030] Ostrom E . 2009. A general framework for analyzing sustainability of social‐ecological systems. Science 325:419–422. 19628857 10.1126/science.1172133

[cobi13632-bib-0031] Özesmi U , Özesmi SL . 2004. Ecological models based on people's knowledge: a multi‐step fuzzy cognitive mapping approach. Ecological Modelling 176:43–64.

[cobi13632-bib-0032] Pagano A , Pluchinotta I , Pengal P , Cokan B , Giordano R . 2019. Engaging stakeholders in the assessment of NBS effectiveness in flood risk reduction: a participatory system dynamics model for benefits and multibenefits evaluation. Science of the Total Environment 690:543–555. 10.1016/j.scitotenv.2019.07.059 31301495

[cobi13632-bib-0033] Perry CT , et al. 2012. Estimating rates of biologically driven coral reef framework production and erosion: a new census‐based carbonate budget methodology and applications to the reefs of Bonaire. Coral Reefs 31:853–868.

[cobi13632-bib-0034] Pinch TJ , Bijker WE . 1984. The social construction of facts and artefacts: or how the sociology of science and the sociology of technology might benefit each other. Social Studies of Science 14:399–441.

[cobi13632-bib-0035] Pluchinotta I , Esposito D , Camarda D . 2019. Fuzzy cognitive mapping to support multi‐agent decisions in development of urban policymaking. Sustainable Cities and Society 46:101402.

[cobi13632-bib-0036] Snijders L , Blumstein DT , Stanley CR , Franks DW . 2017. Animal social network theory can help wildlife conservation. Trends in Ecology & Evolution 32:567–577. 28648805 10.1016/j.tree.2017.05.005

[cobi13632-bib-0037] Tuck ME , Kench PS , Ford MR , Masselink G . 2019. Physical modelling of the response of reef islands to sea‐level rise. Geology 47:803–806.

[cobi13632-bib-0038] Van den Bossche P , Gijselaers W , Segers M , Woltjer G , Kirschner P . 2011. Team learning: building shared mental models. Instructional Science 39:283–301.

[cobi13632-bib-0039] Verburg PH , et al. 2016. Methods and approaches to modelling the Anthropocene. Global Environmental Change 39:328–340.

[cobi13632-bib-0040] Voinov A , et al. 2016. Modelling with stakeholders – next generation. Environmental Modelling & Software 77:196–220.

[cobi13632-bib-0041] Voinov A , et al. 2018. Tools and methods in participatory modeling: selecting the right tool for the job. Environmental Modelling & Software 109:232–255.

[cobi13632-bib-0042] Werhane PH , Hartman LP , Moberg D , Englehardt E , Pritchard M , Parmar B . 2011. Social constructivism, mental models, and problems of obedience. Journal of Business Ethics 100:103–118.

[cobi13632-bib-0043] Wood MD , Bostrom A , Convertino M , Kovacs D , Linkov I . 2012. A moment of mental model clarity: response to Jones et al. 2011. Ecology and Society 17. 10.5751/ES-05122-170407.

