# Supplementary material for: Developing shared qualitative models for complex systems
Source: Conserv Biol. 2020 Dec 21;35(3):1039–50. doi: 10.1111/cobi.13632 (PMC8317195; doi:10.1111/cobi.13632)
Supplement: Supplementary file 1 — Supporting Information [file COBI-35-1039-s003.docx]

# Appendix S1: Supplementary materials describing additional steps and providing additional details for creating a shared qualitative model of a complex system

Full details for Steps 1.3, 1.4, 2.1, 2.3, 3.2 and 3.4 provided. Additional materials provided, in support of the text in the main manuscript, for Steps 1.1. 2.2, 3.1, 3.3, 4b.2, 4b.3 and 4b.5 (see Fig. 1).


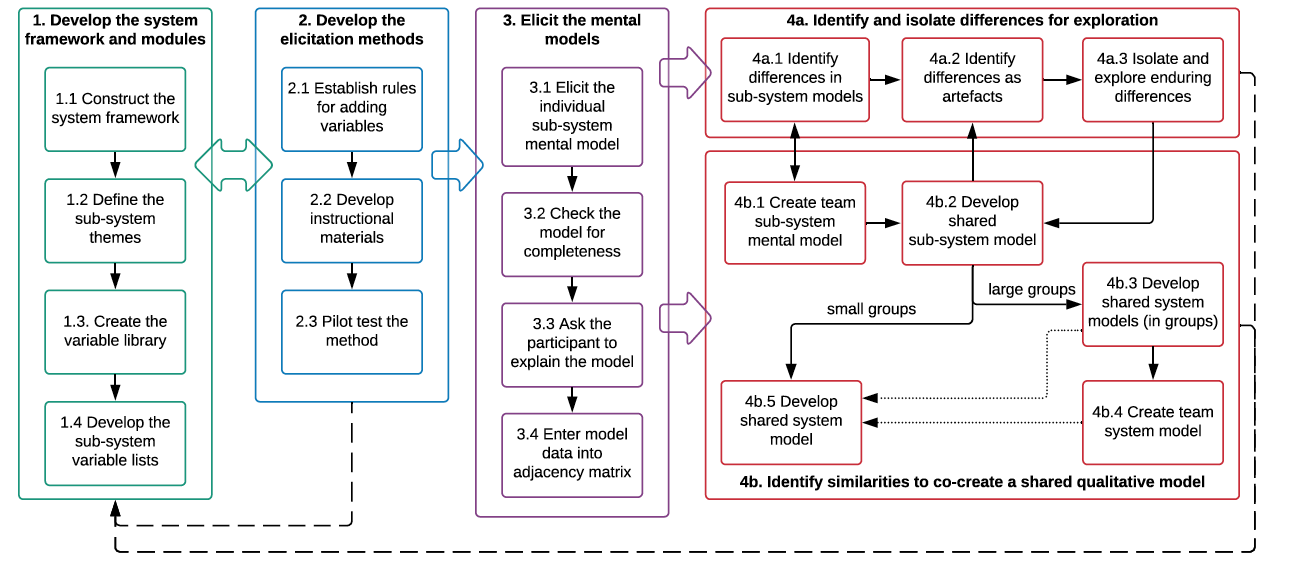


**Figure 1:** Overview of the method for creating a shared qualitative model of a complex system, comprising four phases.

# Phase 1: Develop the system framework and sub-system modules

## Step 1.1: Construct the system framework of the complex system

**Table S1:** Criteria for developing modules of the complex system

| **Module** | **Dominant process (P) or system element (E)** | **Number of variables** | **Dominant modelling component** | **Dominant driver** | **Module output** |
| --- | --- | --- | --- | --- | --- |
| 1. Calcification | Calcification (P) | 28 | Coral and CCA calcification | Ocean acidification and temperature | Total calcification rate* |
| 2. Coral community carbonate production | Coral community (E) | 36 | Coral cover and/or carbonate production | Cyclone and bleaching | Total rate of calcification* |
| 3. Reef accretion | Reef accretion (P) | 27 | Reef accretion | Sea level rise | Reef accretion* |
| 4. Bioerosion | Bioerosion (P) | 24 | Bioerosion | Environmental drivers | Carbonate removal |
| 5. Carbonate sediment production | Carbonate sediments (E) | 28 | Sediment production | Environmental and physical drivers | Net carbonate sediment production* |
| 6. Reef Island | Island change (P) | 22 | Island characteristics | Reef hydrodynamics | Island height, area and shape** |

*these are carbonate sinks.

## Step 1.2: Define the sub-system module themes

Refer to manuscript for details

## Step 1.3: Create the variable library

Once the system framework has been constructed, the main variables that influence the processes and elements within each sub-system module can be identified and compiled to develop a variable library. The variable library includes all known and potential variables required to elicit a mental model for each of the sub-system modules (Phase 3), including definitions and associated units (Table 1, manuscript). In a biological model, these variables are typically a combination of ecological and environmental influencers. At this stage, it is not necessary to consider the influences between variables, but to ensure that all variables that are needed for a functional systems model are included for the modelling process (Phase 2 and 3). The construction of the variable library will require a literature review and potentially some consultation with knowledge experts.

Each variable needs to be clearly defined to ensure that participants interpret them in the same way. Definitions should include a brief explanation of the variable and associated unit (if applicable). Because each variable should be familiar to the participants, definitions and units should be largely based on the most accepted and commonly documented interpretation of that variable, where possible.

## Step 1.4: Develop the module-specific variable lists

Once the system framework is broken into sub-system modules, the ‘variable library’ can be used to develop ‘variable lists’ for each module. The ‘variable list’ needs to include all variables that may be used by the participant in eliciting their mental model, without overwhelming them. The participant needs to have sufficient knowledge of the influences within that sub-system module – the more variables (i.e. complexity) the lower the likelihood of their confidence in their knowledge of all influences. If the variable list contains more than 50 variables, it might be necessary to break the module into two or more smaller modules. Alternatively, it might be possible to combine variables to reduce the variable list. Decisions around the validity and reliability of these choices should be documented and justified.

If variables are combined to simplify the elicitation process, it might be necessary to either re-define a well-known variable to suit the modelling process or create a new variable. These new definitions need to be highlighted to the participant prior to the elicitation process. As such, it is important that the variable list be a focus of the pilot testing stage (Step 2.3)) to ensure that all required variables are included and appropriate, and that unambiguous definitions are provided (Bryman 2016).

Offering participants a pre-determined variable list to assist in eliciting their mental model of the sub-system provides opportunities for quantitative comparison of similarities and differences between the individual mental models (see Moon et al. 2019 for a discussion of researcher- versus participant-generated variables). The method also allows for individual models to be aggregated into a team mental model of the sub-system module (see Step 4b.1).

# Phase 2: Develop the elicitation methods

## Step 2.1: Establish rules for including additional variables

If participants include new variables into their mental model, the process of data analysis and the development of the team sub-system model becomes more difficult. Yet, it is important that participants are allowed to introduce new variables, because people see the world in different ways and a need exists to allow participants to express ‘their reality’ (e.g. Jones et al. 2011; Moon and Blackman 2014). In each sub-system module, we encouraged people to add new variables but only if they felt strongly that they could not complete their model without the new variable/s, *and* after fully reviewing the full variable list and definitions. This approach was achievable given that each module represented a functioning unit with clearly a clearly defined process or element, modelling component and output (Table S1). Instances may exist where a variable is defined in several different ways depending on an individual’s knowledge and understanding of that variable. For example, the influence of waves on coral reefs can be described as wave energy, wave climate, wave height and period. These labels all capture slightly different aspects of waves, so whether a participant thinks it is appropriate or not to use a different version of a variable will influence their need to add to the variable list. We recommend, however, that participants limit the number of new variables to fewer than five. Any new variables introduced during individual mental model elicitation should be discussed between participants during the shared sub-system model elicitation process (Step 4b.2).

## Step 2.2: Develop instructional material

Typically, mental models are elicited in person because the method is usually new to participants, and specific relationships in the final model need to be captured during the modelling process in a way that allows them to be input into an adjacency matrix for analysis (see Step 3.4). Due to the participants being located in different international locations, we developed materials to enable self-elicitation of the mental model. We tested the suitability of the materials for ‘remote elicitation’ to ensure that all model data could be successfully input into an adjacency matrix. The decision to elicit mental models remotely should be made on the basis of project resources, the complexity of the module and the participants’ familiarity with model elicitation.

The instructional video provided an example of how to self-elicit a mental model. The video was developed by a social scientist who guided the modelling process and a carbonate sedimentologist who acted as the ‘expert’ in the video. The social scientist explained the task to the expert, who was then supported in eliciting their mental model. The expert elicited their model while the social scientist ensured that the approach was capturing all the necessary data for analysis. For example, each variable had to have at least one influence (denoted by an arrow) coming into or going out of it. The video focused on the computer screen, which was the platform for model development. Models can also be elicited using pen and paper or modelling software.

We asked participants to complete their mental model of the sub-system module in a Powerpoint file. A part of our reasoning here was to develop a method that could be replicated with commonly available resources. This file included all the variables set up in boxes that could be moved around as part of the elicitation process. The modelling materials also included an excel spreadsheet that listed all variables, their associated units and definitions. One tab in the excel spreadsheet provided the variable library and their definitions; another tab provided the module-specific variable list (see Phase 1).

## Step 2.3: Pilot test the method

The pilot test stage requires individuals who have some knowledge of the sub-system. For our study, we asked an expert in the field or a related field for each of the six sub-system modules to assess the method. Specifically, they assessed: 1) the variable library, lists and definitions; 2) the modelling process as outlined in the written instructions and video; and 3) the timeframe required to complete the elicitation. The assessment of the variable list and library included the completeness of the list, the number of variables included and the definition for each variable. As a result of the pilot test, we made a few minor adjustments to each sub-system module variable list (<3), which included the addition of new variables and the clarification of variable definitions. All pilot testers were able to complete the model elicitation within the suggested timeframe. Each model generated by the testers was also assessed to ensure that the exercise had been completed appropriately for data analysis purposes.

# Phase 3: Elicit the individual mental model

## Step 3.1: Elicit the individual sub-system mental model

On completion of the pilot test, the elicitation instructions and materials were distributed to the participants. For each module, we used a purposive sampling strategy (Bryman 2016) to identify four to six experts for each sub-system module group. We invited 38 participants to elicit a model and received a total of 28 mental models; a response rate of 74%. Of the confirmed participants, 21 were university academics (from nine universities and four countries), six were government employees (including science and conservation management) and one was a private environmental consultant. Universities academics (four PhD students with comprehensive knowledge on sub-system module topics, five Post Doctoral Research Fellows, nine Senior Research Fellows, three Associate Professors) made up the bulk of the participants across the six sub-system modules. However, we also wanted to include those individuals with field and management experience, who would likely become the end users of the final carbonate reef system model. Their input into the model development focused on providing real-world examples of these complex systems and useability of the end product.

## Step 3.1: Elicit the individual mental model

Variables could also be grouped using a red box. Any arrow going from one variable into the red box indicates that the variable from which the arrow is coming influences all the variables within the box equally. Likewise an arrow leaving the red box indicates that all variables in the red box are having the same influencing affect on the variables to which they are connected by an arrow. Grouping the variables reduces the number of arrows in the model, but should be flagged for discussion during the interviews (see Steps 3.2 and 3.3) to ensure that they were used unambiguously (see Fig. 3 in the manuscript for an example of an individual sub-system model).

## Step 3.2: Check the model for completeness

On completion of the modelling exercise, participants submitted the Powerpoint file of their model for a quality check. This check involved ensuring that all variables had at least one arrow going into or out of it; red boxes used for grouping variables had been used unambiguously; all arrows had a number for strength and a letter for confidence; and all arrows were unambiguous in their starting point and destination. Further, any new variables introduced were noted for discussion. If any of the above quality checks were not met, the participant was contacted and asked to amend their model so it complied with the instructions. Compliance was essential for model data input, analysis and comparison.

## Step 3.3: Ask the participant to explain their model

**Table S2:** Post-elicitation interview schedule

| **Introduction**  The interview comprises 15 questions. The interview should take about 30 to 45 minutes of your time. The data will be used for to inform the development of the workshop and might be used in academic papers, although any information will be non-identifiable. The interview will be recorded, unless you specify otherwise, and you are welcome to ask me to stop recording, or stop the interview at anytime.   - Do you give your consent to participating?   **Section A: Overview of modelling process**   1. How did you find the modelling process? 2. Have you carried out this type of modelling before either informally or formally? If so, how does it compare as a modelling method? 3. How long did he take you to complete the model in total?    1. <1hr, 1-3hr, 3-5hr, 1 day, 1 day+ If more than one day, how many days 4. Did you use external resources? If so, what did these include?    1. Own published    2. Own unpublished    3. Others published    4. others unpublished    5. material seen at conferences    6. reports    7. anecdotal    8. experience    9. best guess    10. other   **Section B: Model development and data checking**   1. Could you please talk me through your model? 2. How did you go about determining your own level of confidence in your knowledge of the relationships between variables? 3. Were there any aspect of developing the model that were problematic, if so, what and why? 4. Describe how easy it was to create this type of model i.e. to map out the relationships in a directed graph?   **Section C: Feedback**   1. How might we improve the modelling process? 2. Did you rely more on the written instructions or the video? 3. How did you find using Powerpoint for the modelling process? 4. Was the list of variables provided sufficiently comprehensive and appropriate?   **Section D: Modelling value/benefit**   1. What value and/or benefit has developing the model brought to you, if any? 2. Do you have any ideas or suggestions about how you think it would be useful to approach the exercise for your group model during the workshop? 3. Do you have any question for me about the process or the upcoming workshop or about the research in general? |
| --- |

## Step 3.4: Enter model data into adjacency matrix

All data from individual models were transferred into a data adjacency matrix (see Appendix S3 for detailed instructions on how to input data) in excel (Langan-Fox et al. 2001). Once all individual models for one sub-system module had been transferred to an adjacency matrix, the individual matrices were merged to create two (strength and confidence) sub-system module matrices (Appendix S3). To capture and highlight links that had higher strength and confidence, we multiplied the strength matrix by the confidence matrix and applied a heat map to the final values (Appendix S3). We also created a data matrix that calculated the number of models representing that link (as opposed to the strength/confidence rating; Appendix S3). These data matrices were used to create the team sub-system mental models that were used in the workshop (Step 4b.1).

# Phase 4: Exploring similarities and differences

The development of the shared qualitative system model was conducted during a two-day workshop in Perth, Australia in November 2019. Of the 28 participants, 21 were able to attend the workshop and four were able to log in remotely for approximately three hours per day, with one participant working remotely for the whole day. The 25 participants were relatively evenly spread between the six modules. There was also only one remote conversation per module (for four modules). The first day of the workshop focused on creating the shared sub-system model of each module from the team sub-system models, involving only those participants who had created a model for that module (i.e. all Module 1 participants were grouped together). The second day focused on creating the qualitative shared system model, which was conducted in multi-disciplinary groups, with one member from each module in one of three groups.

Each day started with a 30-minute presentation that outlined the day’s activities, centring around three tasks, and finished with a group module presentation on Day 1, providing the opportunity for participants working on the other modules to ask questions and make suggestions, and an open forum on Day 2 (Table S).

**Table S3:** Example structure of shared qualitative system modelling workshop

| **Day 1** |  |
| --- | --- |
| 08:30 - 09:00 | **Introduction to the shared sub-system modules** |
| 09:00 - 10:30 | Task 1: Sub-system module groups discuss dominant connections in the team sub-system mental model |
| 10:30 – 11:00 | Break |
| 11:00 - 12:30 | Task 2: Sub-system module groups develop the shared sub-system mental model |
| 12:30 – 13:30 | Lunch |
| 13:30 - 15:00 | Task 3: Sub-system module groups finalise shared sub-system mental model and prepare presentation back to the group |
| 15:00 – 15:30 | Break |
| 15:30 - 17:00 | Group presentations |
| **Day 2** |  |
| 08:30 - 09:00 | **Complex system model – how to bring it all together** |
| 09:00 to 10:30  10:30 – 11:00 | Task 1: Shared qualitative model groups focus on variable definitions and driver variables  Break |
| 11:00 - 12:30 | Task 2: Shared qualitative model groups focus on model dependent and linkage variables |
| 12:30 – 13:00 | Lunch |
| 13:00 to 15:00 | Task 3: Qualitative model groups finalise shared model |
| 15:00 – 15:30 | Break |
| 15:30 to 17:00 | **Open forum:** **What next? Key deliverables** a discussion around paper outputs and management tools |

# Phase 4a: Identify and isolate differences for exploration

## Step 4a.1: Identify differences between individuals

Refer to manuscript for details

## Step 4a.2 Identify differences as artefacts

Refer to manuscript for details

## Step 4a.3: Isolate and explore enduring differences

Refer to manuscript for details

# Phase 4b: Identify similarities (and explore differences) to co-create a shared qualitative model

## Step 4b.1: Create team mental model of module (pre-workshop)

Refer to manuscript for details

## Step 4b.2: Elicit shared model of sub-system module

Refer to manuscript for details

**Table S4**: Sample of prompting questions arising from the team sub-system mental model to support the development of the shared sub-system model.

| ***Team model 1: only those influences identified by three or more participants***   1. Check that you agree on all influences shown in Model 1 2. Discuss the wave variables. At the moment wave energy and height are in the model – is this appropriate? 3. Consider how we measure suitable substrate cover? How can this be ‘calculated’ for a model? 4. Bioerosion was a new variable introduced by three of the modellers. Bioerosion is a module, so do you consider that this is where this module would ‘slide’ in here?   ***Team model 2: influences identified by two or more people***   1. Following on from discussions of wave climate/energy etc., do you consider all the lines coming out the wave variables are necessary? 2. One of the issues with this current model is whether or not people have broken down benthic cover into some of the following categories (e.g. living cover, coral composition, branching, foliose, massive etc.), resulting in several different lines between these variables. Discuss the most relevant groupings in regards to quantifying the influence of changing sea level and wave energy on coral community and resulting carbonate production and reef accretion. 3. Does calcification result in measured reef accretion?   ***Team model 3: all influences that had both a high strength and high confidence across all the individual models***   1. Consider all the pathways from coral recruitment through to calcification rate – can these pathways be simplified to reduce potential redundancy? 2. Discuss the influence of rubble cover in the model. Is this variable captured effectively? 3. Evaluate the sediment parameters in the model. Are they effectively related? 4. Consider how accommodation space influences calcification rate? Is it linked exclusively to light and/or wave energy? If so, does the model reflect this relationship already? Can the direct link between accommodation space and calcification rate be removed? 5. Does the degree of mechanical erosion depend on the coral composition? If so, does the model reflect this relationship? 6. Discuss the variable ‘potential reef area’. Consider how this variable can be predicted and then incorporated into a predictive model. |
| --- |

**Table S5:** Example data collection sheets

| **Document model development** | | | |  |  |
| --- | --- | --- | --- | --- | --- |
| **No.** | **Influencer** | **Influenced** | **Change** | **Comments** | |
|  | *Variable with outward influence* | *Variable with inward influence* | *Describe what change was made/not made i.e. document all influences even if no change was made and particularly if there was a discussion around this link* | *Provide comments that summarise group discussions around this influence* | |
| 1 |  |  |  |  | |
| **Document supporting sources for model development** | | | |  |  |
| **No.** | **Influencer** | **Influenced** | **Source** | **Type of information** | **Comments** |
|  | *Variable with outward influence* | *Variable with inward influence* | *These sources would include papers, books, reports as well as expert knowledge from observations, discussions etc.* | *Examples would include raw and worked up data, empirical equations/relationships, theory etc.* | *Any additional information necessary to interpret these sources* |
| 1 |  |  |  |  |  |

## Step 4b.3: Elicit shared qualitative models (in groups)

**Table S6:** A summary of the three different models developed during the team shared qualitative model development (Step 4.3b). Each group took different steps (1 to 8) to develop their models. There was one step that was similar across all models (dark grey; Step 1) and three additional steps (light grey; steps 2 to 4) similar between Groups 2 and 3.

|  | **Model 1** | **Model 2** | **Model 3** |
| --- | --- | --- | --- |
| **Model description** | Model focused on drivers and linkages that were common to 2+ sub-system modules | Model focuses on drivers and plugs in sub-system models | Model incorporates all variables |
| **Advantages** | 1. Less complex, 2. Most influential driver and linkage variables are highlighted both by inclusion but also through variable layering, 3. Involved detailed discussions around driver and linkage variables | 1. Least complex model, 2. Colour coding driver occurrence provides striking visual representation on driver importance | 1. Includes all the variables from all six sub-system model, 2. Includes all links identified among the six sub-system models |
| **Disadvantages** | 1. Not all variables from all six sub-system modules are included | 1. Not all variables from all six sub-system model are included 2. Where the driver influence feeds into each sub-system models is unclear | 1. Most complex model |
| **1** | Identify drivers and cross-reference between sub-system models | Identify drivers and cross-reference between sub-system models | Identify drivers and cross-reference between sub-system models |
| **2** | If driver variable only common to one sub-system module, driver classification was reassessed | Coloured magnets were used to denote which sub-system model the variable was associated with and layered up on the board | Coloured magnets were used to denote which sub-system model the variable was associated with and layered up on the board |
| **3** | Identified linkages and cross-referenced between sub-system models | The number of layers per variable were totalled up and converted to a colour (e.g. blue = 1 sub-system model, green = 2 sub-system models, white = 3 sub-system models etc.). These new colours replaced the previous sub-system model colour coding. | The number of layers per variable were totalled up and converted to a colour (e.g. blue = 1 sub-system model, green = 2 sub-system models, white = 3 sub-system models etc.). These new colours replaced the previous sub-system model colour coding. |
| **4** | If linkage variable only common to one sub-system model, linkage classification was reassessed | Links between drivers were added to the model using green lines on the board | Links between drivers were added to the model using green lines on the board |
| **5** | Removed all variables (including drivers, linkages and dependents) that only occurred in one sub-system model | Represent each module by a variable on the board (e.g. Module 1 Reef accretion) and use on colour to denote these new 'variables' | Identify linkages and cross-reference between sub-system models |
| **6** | Remaining variables were used to develop model using colours to denote associated sub-system model (resulting in copies of variables layered up the board) | Plug' these sub-system model variables into the driver variables already on the board using black lines. | Total the number of layers per linkage variable and convert to a new colour as described in Step 3 |
| **7** | Similar variables were grouped | To facilitate the integration of sub-system model variables into the complex system model, it maybe necessary to identify additional linkage variables between drivers and the sub-system model variables | Add in new links between all variables on the board using a different colour white board marker |
| **8** | Links between drivers and linkages were added to the model using black, green and red lines (as described in section 4b.2) |  | Repeat Steps 5 to 7 with the dependent variables |

## Step 4b.4: Create a team system model

Refer to manuscript for details

## Step 4b.5: Develop the shared qualitative model

**Plate 1:** Photograph of participants engaged in the development of the shared qualitative model.
