# Supplementary material for: Developing shared qualitative models for complex systems
Source: Conserv Biol. 2020 Dec 21;35(3):1039–50. doi: 10.1111/cobi.13632 (PMC8317195; doi:10.1111/cobi.13632)
Supplement: Supplementary file 2 — Supporting Information [file COBI-35-1039-s001.docx]

**Appendix S2: Modelling instructions**

This modelling exercise has been designed to inform a conceptual model to ***future forecast island response to climate change***. This conceptual model is comprised of six individual modules. You have been assigned one of these modules, and we are seeking your expert knowledge on informing the modelling of the individual module.

You are to create a model on only one of the following modules. Each module has a key component for modelling (in bold) and a key driver identified (underlined). You will need to consider how your key driver directly and indirectly influences your key component. Most carbonate budgets function on an annual time-step, so you will need to include all processes that occur over a year or longer.

1. Assessing how a **coral community and reef growth** responds to sea level change
2. Assessing cyclone and bleaching impacts on coral reef **community composition and carbonate production**
3. Assessing the influence of ocean acidification and rising sea surface temperature impacts on **coral and CCA calcification**
4. Assessing reef and environmental drivers of **bioerosion** on coral reefs
5. Assessing biological and physical drivers of **sediment production** (indirect and direct) and **loss** on coral reefs
6. Assessing reef and island factors that influence **reef island maintenance and stability**

For each conceptual model to be successfully collated with other models on the same topic (i.e. module), and then integrated into the overall model, they need to be developed according to the same guidelines. To guide you through the modelling exercise we have provided an instructional video and some specific guidelines below.

1. You have been provided with a list of variables for your module. These variables are found in the Excel file. You are to use as many or as few of these variables as you like in your model. Carefully go through all the provided variables and refer to the variable definition sheet if required.
2. You have also been provided with a Powerpoint file. Open this file. In here you will see that there are 2 sheets. In the first sheet you will see all your variables in boxes (variable page) and in the second sheet a blank page with your module title in red (model page).
3. The variables you include in your model should be based on whether you think each variable is ***important to your module*** rather than whether sufficient data exists that justifies its inclusion. Remember your model is how you **perceive** the relationship between variables, if you think a variable should be included, but you’re not exactly sure how it influences another variable, it is still OK to include it.
4. Select variables that you want to include in your model from the variable page and copy and paste into the model page. Once you are happy with your variable selection, you can now start to think about how they influence one another, the order and the model output.
5. On the model page you can start to move boxes around and consider which ones influence others and the order of flow. At this stage you might want to consider where the end output variable of your model sits relative to the other variables; this variable directly meets the module goal (see above).
6. To link your variables you will need to add an arrow e.g. if variable X influences variable Y, then the arrow must go from variable X to variable Y. All variables in your model must have at least one arrow going into or out of it. A variable can have multiple arrows coming in and out. These influences could be either positive and negative. However, we do not ask you define influences in these terms, only that there is an influence.
7. If you feel that there are some variables that can be grouped together, then you can draw a red box around them. But you need to make sure that you consider that all arrows going into the red box have an equal effect on all variables grouped together, and all arrow/s coming out of the red box demonstrates that all variables in the red box have an equal influence on the next connected variable. If one of the variables in the red box has a separate influence on another variable, make sure that an arrow comes from this variable only (see instructional video for a worked example).
8. Please make sure that your arrows only join variables to other variables and not to other lines. That is, you cannot have arrows that merge together.
9. If you cannot complete your model without the addition of one or more variables, please add these to your model. But only include additional variables if absolutely necessary because the more changes that are made to the variable list, the reduced compatibility between models in the module.
10. Once you have completed the model, can you then grade each of the arrows from 1 (weak influence) to 5 (strong influence) to indicate the strength of the influence e.g. a value of 5 between X and Y suggests that variable X has a very strong influence on variable Y. These gradings just refer to the strength of the relationship.
11. We also ask you to indicate the degree of confidence in your knowledge of the influence using letter A to C. The letter A indicates you have a weak confidence in your knowledge of that influence, the letter B indicates you are moderately confident and the letter C indicates that you are very confident.
12. During the modelling process you are free to check the literature as much as you want or need to, but we do not expect this modelling process to take too much of your time. The aim is to capture your current understanding of the system as well as highlight any potential knowledge gaps, both of which will be discussed further in the workshop.
13. Feel free to make notes in the text space below the model
14. The submitted model will be checked to ensure that all data has been provided in a way that enables analysis. To help with this process, we ask you to quickly run through the checklist on the next page.

These guidelines are intentionally broad to prevent constraining your model and biasing the model outputs. If, however, you would like additional input before creating your model, please contact Dr Nicola Browne.

***What next?***

Once all attendees have submitted their models and graded variables, the data will be checked for completeness and combined for analysis. We will arrange a time to call you and ask you to explain your model so that we can be sure we have captured all the relevant data and knowledge. This phase of the data collection process will also start to build a picture of how each person has gone about developing their model and where opportunities for knowledge sharing exist.

**Checklist**

Please make sure you have completed the following:

1. You have watched the accompanying instructional video
2. All your variables in your model have at least one arrow going into or out of the box
3. All arrows in your model are clearly labelled with a number (1 to 5) AND letter (A to C)
4. If you have grouped variables using a red box, make sure that all arrows going into and out of the red box are clear (as described in point 7 above and in the instructional video).
5. Ensure that you have understood the definitions of all variables used in the model by referring to the variable definition sheet
6. If you have created another variable in your model (or used another one from the variable definition sheet), please confirm your definition of this variable. We also ask that you do not create too many more variables as explained above.
7. Double check that you model addresses the module topic you were assigned.
